# Combining T2Bacteria and T2Candida Panels for Diagnosing Intra-Abdominal Infections: A Prospective Multicenter Study

**DOI:** 10.3390/jof8080832

**Published:** 2022-08-09

**Authors:** Anders Krifors, Måns Ullberg, Markus Castegren, Johan Petersson, Ernesto Sparrelid, Volkan Özenci, Ola Blennow

**Affiliations:** 1 Department of Physiology and Pharmacology, Karolinska Institutet, 14186 Stockholm, Sweden; 2 Centre for Clinical Research Västmanland, Uppsala University, 75236 Uppsala, Sweden; 3 Department of Clinical Microbiology, Karolinska University Hospital, 14186 Stockholm, Sweden; 4 Perioperative Medicine and Intensive Care (PMI), Karolinska University Hospital, 14186 Stockholm, Sweden; 5 Department of Clinical Science, Intervention and Technology (CLINTEC), Karolinska Institutet, 14186 Stockholm, Sweden; 6 Department of Laboratory Medicine, Division of Clinical Microbiology, Karolinska Institutet, 14186 Stockholm, Sweden; 7 Department of Infectious Diseases, Karolinska University Hospital, 14186 Stockholm, Sweden

**Keywords:** T2 magnetic resonance, T2Bacteria, intra-abdominal infection

## Abstract

The T2Bacteria panel is a direct-from-blood assay that delivers rapid results, targeting *E. coli*, *S. aureus*, *K. pneumoniae*, *A. baumanii*, *P. aeruginosa*, and *E. faecium* (ESKAPE pathogens). In this study, T2Bacteria and T2Candida (targeting *C. albicans*/*C. tropicalis*, *C. glabrata*/*C. krusei*, and *C. parapsilosis*) were evaluated in parallel with blood cultures in 101 consecutive surgical patients with suspected intra-abdominal infection admitted to the intensive care unit or high dependency unit. Fifteen patients had bacteremia, with T2Bacteria correctly identifying all on-panel (*n* = 8) pathogens. T2Bacteria was positive in 19 additional patients, 11 of whom had supportive cultures from other normally sterile sites (newly inserted drains, perioperative cultures or blood cultures) within seven days. Six of these eleven patients (55%) received broad-spectrum antibiotics at the sampling time. T2Candida identified the two cases of blood-culture-positive candidemia and was positive in seven additional patients, three of whom were confirmed to have intra-abdominal candidiasis. Of four patients with concurrent T2Bacteria and T2Candida positivity, only one patient had positive blood cultures (candidemia), while three out of four patients had supporting microbiological evidence of a mixed infection. T2Bacteria and T2Candida were fast and accurate in diagnosing on-panel bloodstream infections, and T2Bacteria was able to detect culture-negative intra-abdominal infections.

## 1. Introduction

Intra-abdominal infections (IAI) are frequent among surgical patients in the intensive care unit (ICU) and are associated with significant morbidity and mortality [1]. A prompt diagnosis, source control, and adequate antibiotic or antifungal therapy remain cornerstones in IAI management [2]. However, blood cultures have been reported to be positive in only 19% of patients with postoperative IAI [3], and it may not be possible to obtain access to a drainage or perioperative culture due to the critical state of the patient. In addition, IAIs are often polymicrobial, and the potential growth of both bacteria and *Candida* spp. is a challenge to culture-based methods because the higher growth rate of bacteria may hide the presence of *Candida* spp. [4]. Concomitant bacteremia has been reported in 20% to 38% of candidemia episodes [5,6]. Selective blood culture bottles, such as mycosis bottles, have been reported to improve the detection of candidemia in polymicrobial bloodstream infections by inhibiting bacterial growth [7]. However, the overall sensitivity of culture-based diagnostics is low in detecting monomicrobial bloodstream infections and even lower in polymicrobial bloodstream infections. Therefore, molecular tests can be attractive complements to culture-based methods and offer the additional advantages of easy handling and rapid results [8].

T2Bacteria and T2Candida assays detect bacterial and *Candida* DNA in whole blood samples within 3 to 5 h using T2 Magnetic Resonance (T2MR) technology [9]. The system is fully automatic and works by amplifying cell-bound *Candida* or bacterial DNA by PCR followed by hybridization to create clusters of magnetic nanoparticles detected by magnetic resonance changes. The T2Bacteria panel includes the ESKAPE pathogens (*E. coli*, *S. aureus*, *K. pneumoniae*, *A. baumanii*, *P. aeruginosa*, and *E. faecium*), bacteria with specific clinical challenges related to virulence and multidrug resistance that constitute around half of all bloodstream infections [9,10]. T2Bacteria has been reported to provide high sensitivity (90%) and specificity (90%) for on-panel bloodstream infections. However, studies of the performance of T2Bacteria in IAI, where seeding to the bloodstream may be intermittent, are lacking [9]. Besides providing fast microbiological results in IAI patients with bacteremia, T2Bacteria can potentially increase the diagnostic yield in culture-negative IAI since it can remain positive after empiric antibiotic therapy has been initiated [11].

T2Candida can detect the five most clinically relevant *Candida* spp. (reported as *C. albicans*/*C. tropicalis*, *C. glabrata*/*C. krusei*, and *C. parapsilosis*). Previous studies have reported the use of either T2Bacteria or T2Candida assays, but no prospective clinical studies on the combined use of these panels are available. Recently, we reported the results of a prospective study evaluating the performance of T2Candida in intra-abdominal candidiasis (IAC) [12]. A subset of these patients was also tested simultaneously with T2Bacteria, providing a unique opportunity to evaluate the performance of combining T2Bacteria and T2Candida in potential polymicrobial IAI infections.

## 2. Materials and Methods

This prospective observational multicenter study was conducted at the ICU and surgical high dependency unit of the Karolinska University Hospitals, Huddinge and Solna, Stockholm, and the ICU at Västerås Hospital, Västerås. Prospective patients older than 18 years, with preceding gastrointestinal surgery or necrotizing pancreatitis who were admitted to the ICU and surgical high dependency unit between May 2019 to February 2021 and who underwent diagnostic blood cultures per the attending physician’s request, were simultaneously assessed with T2Bacteria and T2Candida panels. Blood cultures were analyzed at the microbiology department at each study center using BacT/ALERT FA Plus Aerobic and FN Plus Anaerobic media (bioMérieux, Marcy l’Etoile, France) and incubated in the BacT/ALERT VIRTUO Culture System (bioMérieux, Marcy l’Etoile, France) for a maximum period of 10 days.

Positive blood cultures were subjected to Gram stain microscopy, sub-culturing, and analysis using direct-matrix-assisted laser desorption/ionization–time-of-flight mass spectrometry from single spotted colonies.

T2Bacteria and T2Candida analyses were performed using 3 mL of whole blood collected in EDTA vacutainers and loaded into the fully automatic T2Dx instrument (T2Biosystems, Lexington, KY, USA) as per the manufacturer’s instructions. If the internal control failed, the sample was reported as invalid. All results were made clinically available.

The T2Bacteria and T2Candida results were compared to the accompanying blood cultures and drainage/perioperative cultures obtained within seven days of sampling. Medical charts were reviewed retrospectively for baseline characteristics, clinical data, 30-day mortality, and antibiotic and antifungal treatment.

The study was approved by the Swedish Ethical Review Board (DNR 2019-00603).

## 3. Results

In total, 101 consecutive patients with preceding gastrointestinal surgery (*n* = 86, 85%) or necrotizing pancreatitis (*n* = 15, 15%) were included in the study. The median age of the patients was 67 years, and 56% (*n* = 57) of the cohort were male (Table 1). The mean Sequential Organ Failure Assessment Score was 3.6, and the 30-day mortality was 13% (*n* = 13). Bacteremia was present in 15 patients, 8 of which were pathogens on the T2Bacteria panel (Table 2). T2Bacteria was positive in 27% (*n* = 27) of the patients, including all eight cases of on-panel bacteremia. Of the remaining 19 T2Bacteria-positive patients, 11 patients had corresponding findings from invasive cultures obtained within seven days from the positive T2Bacteria test (blood cultures (*n* = 3), perioperative cultures (*n* = 2), or cultures from newly placed (<24 h) drains (*n* = 6). Of the remaining eight T2Bacteria-positive patients, one patient had findings of the corresponding bacteria in ascites more than seven days later. All but one of the eight patients were clinically assessed as having an IAI.

Thirteen of twenty-seven (48%) T2Bacteria-positive patients were receiving broad-spectrum antibiotics at the time of sampling, including ten out of the nineteen discordant cases (positive T2Bacteria/negative blood culture). Out of the 27 T2Bacteria-positive patients, 4 were receiving inadequate treatment for the pathogen identified by T2Bacteria (four cases of *E. faecium* receiving either piperacillin/tazobactam or meropenem).

T2Candida was positive in 9% (*n* = 9) of the patients. Intra-abdominal candidiasis (IAC) was confirmed in 5/9 patients, with one case of concomitant candidemia. One T2Candida-positive patient had candidemia without IAC and two of the remaining four positive patients were clinically deemed to have IAC.

There were no patients with concomitant bacteremia and candidemia. However, four patients were simultaneously positive in T2Candida and T2Bacteria: (1) *C. albicans/C. tropicalis* and *E. faecium* (confirmed IAC with *C. albicans*, growth of *E. faecium* in blood and drainage cultures nine days later); (2) *C. parapsilosis* and *P. aeruginosa* (IAC not confirmed, growth of *P. aeruginosa* in a drain ten days earlier); (3) *C. albicans/C. tropicalis* and *P. aeruginosa* and *E. coli* (blood cultures positive for *C. albicans*, growth of *P. aeruginosa* in perioperative cultures); (4) *C. albicans/C.* tropicalis and *E. faecium* (not fulfilling IAC criteria because cultures with growth of *C. albicans* were not taken until 48 h after insertion of a new drainage, growth of *E. faecium* in perioperative cultures). Clinically, all four cases were assessed to be mixed IAI with *Candida* spp. and bacteria.

## 4. Discussion

This prospective multicenter study is the first to focus on the performance of T2Bacteria in IAI. International guidelines emphasize the importance of early identification and adequate antibiotic therapy in IAI [2]. However, definite microbiological findings are only available in 70% of patients with IAI [13,14], and more than a third of IAI patients receive ineffective empiric antibiotic treatment [14]. In the study, the T2Bacteria panel provided 100% (8/8) sensitivity for on-panel bacteremia, which is in line with previous studies reporting sensitivities ranging from 83 to 94% [9,15,16]. The ESKAPE pathogens represented 53% (8/15) of the blood isolates, similar to the 48% reported in the T2Bacteria registration study by Nguyen et al. [9]. Importantly, T2Bacteria was positive in 19 blood-culture-negative patients, of which 11 had supporting cultures confirming an IAI. In a study by Kalligeros et al., 21 discordant cases (positive T2Bacteria/negative blood culture) were thoroughly examined, with the finding that 15/21 (71%) of the T2Bacteria results were supported by microbiological findings [11]. The authors also reported that recent use of antibiotics was associated with discordant results. This finding is supported by the results of our study, where 53% (10/19) of patients with discordant results were receiving broad-spectrum antibiotics at the time of sampling. Thus, the T2Bacteria panel can provide valuable microbiological results in IAI, particularly when antibiotic therapy has already been initiated.

Another advantage of T2Bacteria is its fast turnaround time, with a result available in only 3 to 5 h [17]. The ESKAPE pathogens are associated with high mortality, and many isolates are resistant to commonly used empiric antibiotic therapies for IAI [18]. Carbapenem-resistant *Acinetobacter* and Carbapenem-resistant *Enterobacteriaceae* are among the top pathogens on the Centers for Disease Controls’ list of urgent threats [19]. In our study, all cases of *E. faecium* received inappropriate antibiotic therapy at sampling, highlighting the clinical usefulness of a rapid molecular test.

It has been demonstrated that the limit of detection of the T2Dx instrument is as low as 1 CFU/mL in spiked blood cultures [17]. Such a low threshold raises concerns that heavy gut colonization could lead to “false positive” T2 results due to gut wall translocation without the presence of clinical infection. However, only 7 of 27 (26%) of T2Bacteria-positive patients lacked supportive cultures, and all but one was clinically deemed to have IAI. An important reason for this low rate of “false positives” may be that the T2Dx instrument detects only cell-bound DNA and not free DNA [17]. Thus, for a T2 test to become positive, an on-panel bacteria or *Candida* spp. must be present in the blood sample.

In the present study, T2Candida was analyzed in parallel with T2Bacteria. Mixed *Candida*–bacterial bloodstream infections constitute approximately 20% of all candidemia cases [20]. Detecting mixed infections is problematic since culture-based methods have been reported to have high rates of false negativity, making non-culture-based methods necessary. Interestingly, no polymicrobial bloodstream infections were detected, despite four patients having both T2Bacteria and T2Candida positive tests. Based on supportive cultures and the clinical presentation, the positive results were assessed as significant by the treating physicians in all four patients, making it plausible that these cases were indeed polymicrobial bloodstream infections.

T2Bacteria and T2Candida have high sensitivity for on-panel bloodstream infections but are expensive and should not be used indiscriminately. However, in critically ill patients with IAI, where a rapid result may lead to both adjustments in antimicrobial treatment and a decision for surgical intervention, their combined use appears to be valuable.

T2Bacteria targets about 50% of blood culture isolates, and its use is not primarily for de-escalation but to tailor treatment. However, de-escalating empiric vancomycin or linezolid after a negative T2Bacteria result could be an option in selected patients receiving empiric MRSA coverage.

The strengths of this study include the prospective and pragmatic design, and a well-defined study population, providing reliable and valid results. Limitations include a low prevalence of on-panel bacteremia.

In conclusion, in patients with IAI, T2Bacteria is fast and accurate for diagnosing on-panel bacteremia, can identify pathogens in blood-culture-negative patients, and can remain positive after initiating antibiotic therapy. Combining T2Bacteria and T2Candida may be a valuable tool for diagnosing mixed infections in critically ill IAI patients.

## Figures and Tables

**Table 1:** Baseline characteristics, *n* = 101.

Male, *n* (%)	57 (56) P
Age (years), median (IQR)	67 (56–73)
Renal failure, eGFR < 60 mL/min *	10 (10)
Immunosuppression, *n* (%)	20 (20)
Chemotherapy (60%) Solid-organ transplant (20%) High dose corticosteroids ^†^ (5%) Other (15%)	
Preceding gastrointestinal surgery, *n* (%)	86 (86)
Necrotizing pancreatitis, *n* (%)	15 (15)
Multiple gastrointestinal surgery, *n* (%)	14 (14)
30-day mortality, *n* (%)	13 (13)
**Clinical data at testing**	
SOFA score (average), ±SD	3.6 ± 3.4
Vasopressor treatment, *n* (%)	35 (35)
Invasive mechanical ventilation, *n* (%)	26 (26)
Admitted to the intensive care unit (%)	42 (42)
Admitted to the intermediate care unit (%)	59 (59)
Total parental nutrition, *n* (%)	21 (21)
Renal replacement therapy, *n* (%)	11 (11)
Broad spectrum antibiotic therapy, *n* (%)	54 (54)
Antifungal therapy at sampling, *n* (%)	10 (10)

Abbreviations: IQR, interquartile range; eGFR, estimated glomerular filtration rate; SOFA, sequential organ failure assessment; SD, standard deviation. * based on the MDRD formula (186.3 × (s-creatinine/88.4) −1.154 × age−0.203 (× 0.742 for female). ^†^ >20 mg prednisone equivalents a day.

**Table 2:** Performance of T2Bacteria panel for on-panel bacterial species.

Species	BC+/T2B+, *n*	BC+/T2B-, *n*	BC-/T2B+, *n*	Supportive Cultures ±7d (BC-/T2B+)
All	8	0	19	11/19
*E. faecium*	1	0	8	5/8
*S. aureus*	2	0	0	-
*K. pneumonia*	1	0	0	-
*A. baumanii*	0	0	0	-
*P. aeruginosa*	0	0	2 *	2/2
*E. coli*	4	0	10 *	5/10

BC, blood culture; T2B, T2Bacteria.* One patient with T2Bacteria positive for *P. aeruginosa + E. coli.*

## Data Availability

The data presented in this study are available from the corresponding author upon request, and the data are not publicly available due to ethical restrictions.
